# Comparative analysis of production and kinetic assay conditions and parameters for SARS-CoV-2 main protease (Mpro): a systematic review

**DOI:** 10.1007/s11033-026-12966-6

**Published:** 2026-10-30

**Authors:** Matheus Ferroni Schwartz, Aisel Valle Garay, Amanda Araújo Souza, Napoleão Fonseca Valadares, Sonia Maria de Freitas

**Affiliations:** University of Brasilia, Institute of Biological Sciences, Biophysics Laboratory, Brasília-DF, 70910-900 Brazil

**Keywords:** SARS-CoV-2, Mpro, COVID-19, Kinetics, Mpro tag-free production, Mpro purification, Mpro substrates, FRET

## Abstract

The main protease (Mpro) of SARS-CoV-2 plays a crucial role in the viral replication cycle and is a key target for COVID-19 antiviral drugs. In this review, we conducted an extensive bibliographic search on the main challenges and experimental limitations reported in the production and biochemical characterization of the Mpro. A large variability in the kinetic parameters was revealed, which compromises comparisons across various studies and the evaluation of inhibitor efficacy. The studies indicate that these discrepancies are mainly associated with experimental limitations, such as low substrate solubility, optical irregularity due to the inner-filter effect, and variations in the oligomeric state of Mpro. This review summarizes and discusses the different strategies for Mpro expression in *Escherichia coli* and purification, with emphasis on obtaining an N-terminal tag-free enzyme, essential for homodimerization into a highly active form for structural and functional characterization. The main FRET-based substrates used in enzymatic assays were compiled and analyzed, as well as alternative methodologies for assessing Mpro enzymatic activity. Furthermore, the effects of assay conditions, such as buffer composition, pH, and the presence of reducing agents were addressed. The intrinsic structural features of Mpro that impact its catalytic function are discussed, including functional asymmetry in substrate binding and the positive allosteric cooperation between monomers. Overall, this review provides a critical and integrated perspective on the main experimental limitations in the production and characterization of Mpro, applicable to other proteases, contributing to the standardization of approaches and advances in the development of more effective inhibitors.

## Introduction

In December 2019, multiple cases of severe pulmonary infection were reported in Hubei, China, prompting international attention. By early January 2020, the etiological agent was isolated and designated 2019-nCoV (2019 novel coronavirus) by the World Health Organization (WHO). This virus was later classified as Severe Acute Respiratory Syndrome Coronavirus 2 (SARS-CoV-2) [1], and the associated disease was termed Coronavirus Disease 2019 (COVID-19). Despite efforts to contain the outbreak in its region of origin, the high transmissibility of SARS-CoV-2 facilitated the onset of a global pandemic, officially declared by the WHO on March 11, 2020. Since then, COVID-19 has affected more than 200 countries and territories, with over 700 million confirmed cases and approximately 7 million deaths reported worldwide as of October 2025. Although disease control has significantly improved, the potential emergence of variants capable of evading antiviral interventions, vaccine-induced immunity, and natural infection-acquired immunity remains a pressing concern for the scientific community [2–4].

The main protease (Mpro) is considered the main target of COVID-19 antivirals development. Despite its critical importance, Mpro enzymatic studies are characterized by a significant degree of "noise," with reported kinetic parameters such as K_m_ and k_cat_ varying widely across different studies. This review aims to systematically address these discrepancies by identifying the structural and experimental factors that influence Mpro enzymatic activity. Firstly, the structural bases of Mpro activity were analyzed, highlighting its dependency on dimerization and complex allosteric communication between monomers. The current landscape of heterologous expression and purification workflows employed to produce tag-free Mpro, necessary for optimal activity, were summarized. This work evaluates the diverse array of synthetic substrates and experimental conditions, including buffer composition, redox environment, and additive effects that contribute to the observed variability in Mprokinetic profile. We hope that this review will provide a clear and useful overview of the key factors that can influence Mpro activity, distinguishing our work from most published reviews focused predominantly on Mpro inhibitor discovery. Finally, we included a practical checklist for Mpro enzymatic assays, designed to promote experimental standardization and as a guideline to obtain the protease in its optimal active state.

## SARS-CoV-2 viral life cycle

Coronaviruses (CoVs) are enveloped, positive-sense, single-stranded RNA viruses with genomes of approximately 26–32 kb-among the largest known RNA viral genomes [5]. CoVs belong to the family Coronaviridae, order Nidovirales, and are classified into four genera: Alphacoronavirus, Betacoronavirus, Gammacoronavirus, and Deltacoronavirus [6]. To date, seven CoV species have been identified as capable of infecting humans. HCoV-229E, HCoV-OC43, HCoV-NL63, and HCoV-HKU1 are associated with 15–30% of common-cold episodes and rarely cause severe disease [7]. In contrast, SARS-CoV and MERS-CoV were responsible for the 2002 and 2012 epidemics, respectively. SARS-CoV-2 is classified within the order Nidovirales, family Coronaviridae, subfamily Coronavirinae, and genus Betacoronavirus.

**Fig. 1:**
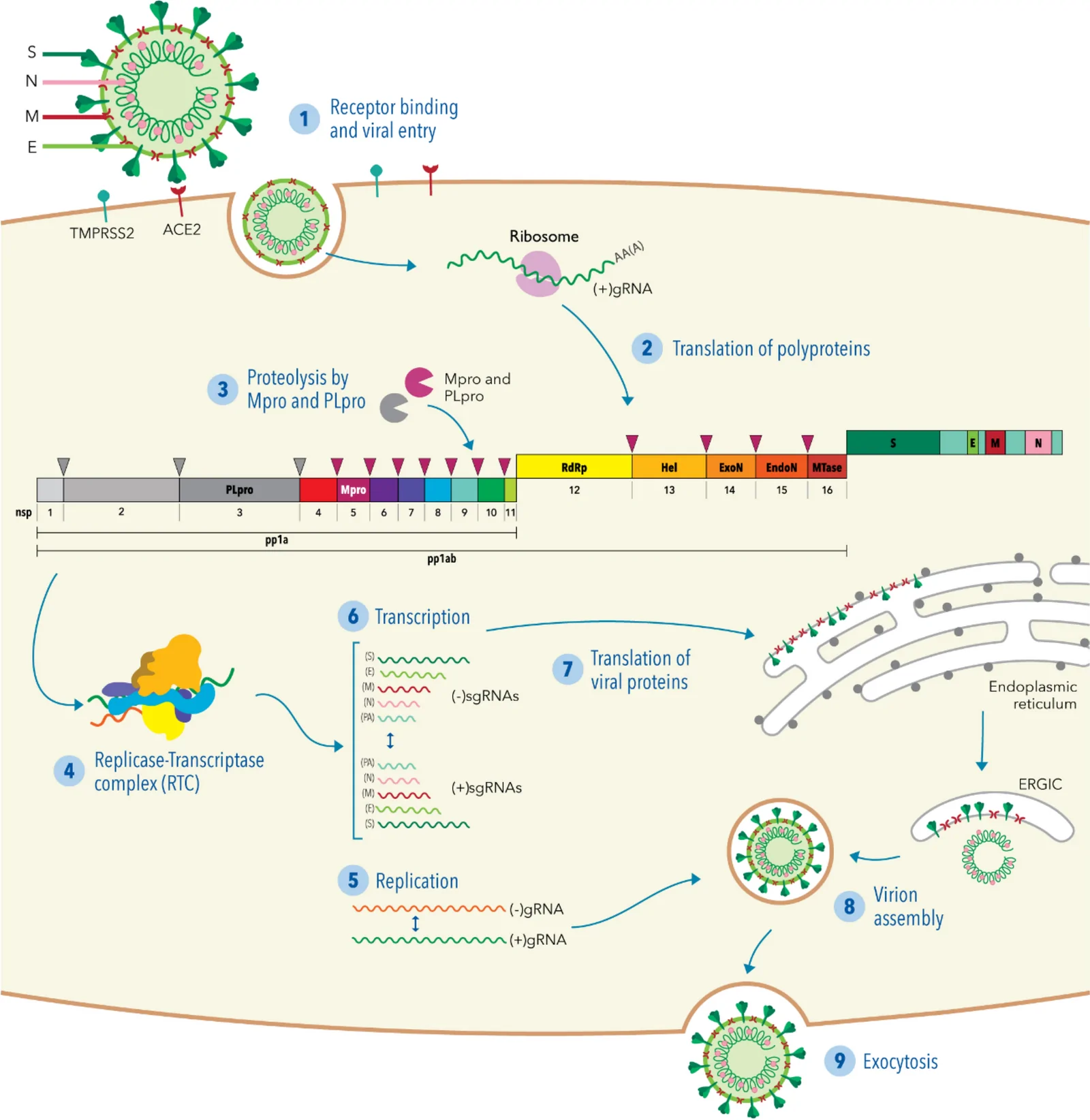
SARS-CoV-2 replication cycle. **(1)** The SARS-CoV-2 viral cycle starts with the binding of the spike (S) protein to ACE2 receptors, followed by conformational changes in S by cellular proteases, mainly TMPRSS2, furin and cathepsin L, which leads to membrane fusion and viral entry. **(2)** The genomic RNA, ( +)ssRNA, is translated into the polyproteins pp1a and pp1ab. **(3)** Viral proteases, PLpro and Mpro, are released from the polyproteins by cis-cleavage events and, subsequently, cleave and release the remaining non-structural proteins (NSPs 1–16). **(4)** The replicase-transcriptase complex (RTC) is formed by NSP6-16. It carries out the **(5)** replication of full-length negative-strand RNA intermediates that act as templates for new genomic ( +) gRNA, and subgenomic RNAs (sgRNAs) by transcription **(6)**. The subgenomic RNA is translated into the structural proteins (S, M, E, and accessory proteins), which are translocated to the endoplasmic reticulum (ER), followed by transit through the Golgi apparatus **(7)**. In the ER-to-Golgi intermediate compartment (ERGIC), the N protein encapsulates the ( +)gRNA and virions are formed **(8)** and subsequently released by exocytosis **(9)**

The SARS-CoV-2 genome contains 14 open reading frames (ORFs). Approximately two-thirds of the genome at the 5′ terminus encodes the replicase polyproteins (ORF1a and ORF1ab), followed by the genes for the structural proteins spike (S), envelope (E), membrane (M) and nucleocapsid (N), interspersed with accessory proteins (Fig. 1). SARS-CoV-2 host cell entry occurs through two primary routes: cell surface entry or the endosomal pathway [8]. The membrane fusion process is driven by conformational changes in the Spike (S) protein induced by binding to angiotensin-converting enzyme 2 receptors (ACE2) and by extracellular protease activity, notably furin, type II transmembrane serine proteases (TMPRSS2), and cathepsin L (for the endosomal pathway).

Following viral entry, ORF1a is translated into the polyprotein pp1a, which contains the nonstructural proteins (NSPs). A stop codon normally terminates translation, yet a −1 ribosomal frameshift triggered by a slippery sequence and an RNA pseudoknot occasionally allows read-through into ORF1ab, producing the pp1ab. The viral polyproteins are cleaved by the papain-like protease (PLpro) and the main protease (Mpro) cysteine-like (3CLpro) to release the NSPs 1–16 [9, 10]. Mpro alone is responsible for the liberation of 11 NSPs, including its maturation by auto-cleavage at both termini. The pp1a-derived NSPs remodel the intracellular environment by suppressing innate immune signaling and inducing the formation of double-membrane vesicles (DMVs) that serve as replication organelles. NSPs 6–16 assemble the replication–transcription complex (RTC) [11, 12]. Within the RTC, the RNA-dependent RNA polymerase (RdRp), NSP12, NSP7, NSP8, and NSP13 as co-factors carry out the replication of full-length negative-strand RNA intermediates that act as templates for new genomic ssRNA(+) and subgenomic RNAs (sgRNAs). Each sgRNA carries a common leader sequence fused to downstream ORFs, enabling expression of S, E, M, and N, together with accessory proteins [13].

## Mpro structure and catalytic mechanism

**Fig. 2:**
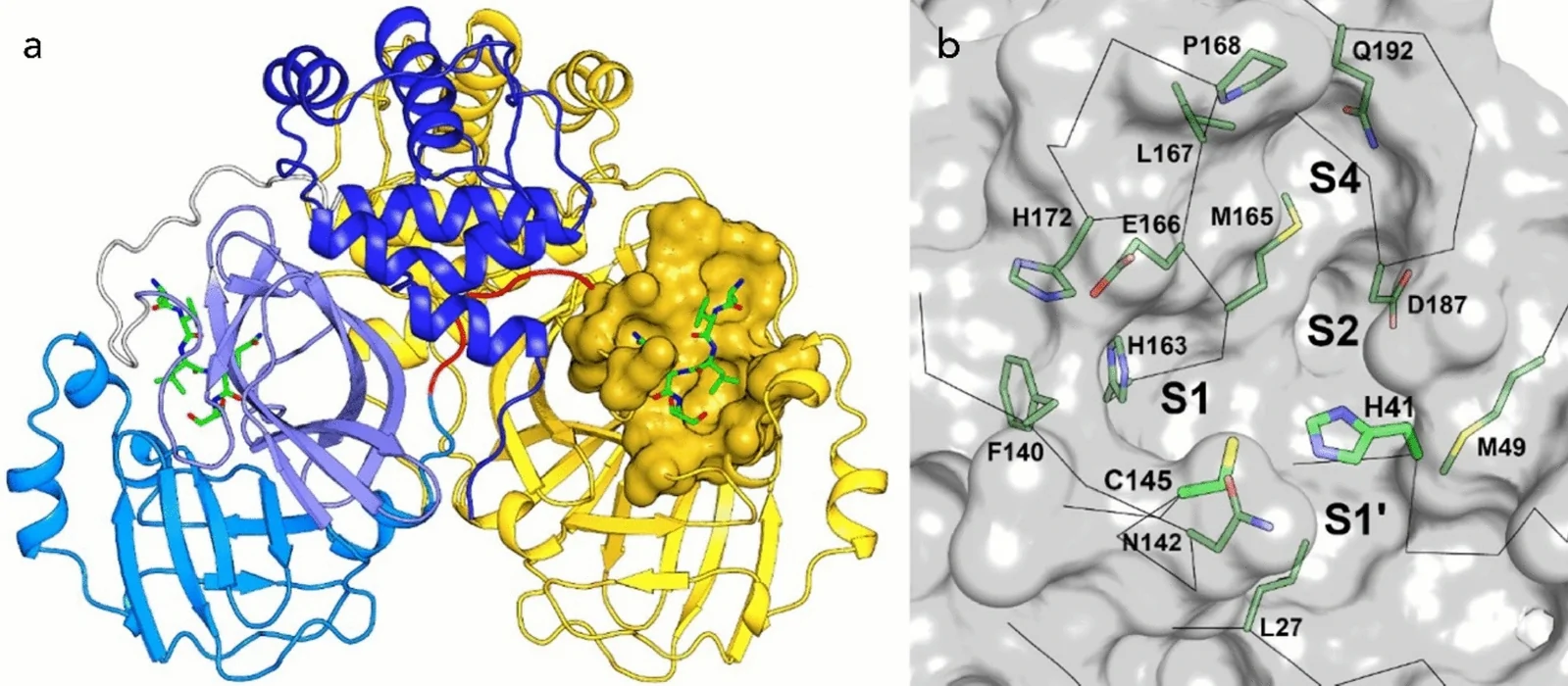
Structural Organization and Active Site Architecture of the SARS-CoV-2 Main Protease (Mpro) homodimer. **(a)** Structure of Mpro homodimer. The left monomer is colored by domain: Domain I is light blue, Domain II is medium blue, and Domain III is dark blue. The inter-domain linker is shown in white, and the N-terminal finger (residues 1–7) is highlighted in red. The monomer on the right is depicted in yellow with the substrate-binding site rendered as a surface. The peptide substrate AVLQS (green sticks) is bound in the active site of each monomer, engaging subsites S4 to S1’. **(b)** Close-up view of the empty substrate-binding cleft, highlighting four subsites, the catalytic dyad (His41 and Cys145), and additional residues shaping the active site architecture. Made with PyMOL, PDB IDs 9EYA and 6LU7

SARS-CoV-2 main protease (Mpro; EC 3.4.22.69) is a C30 family, clan-PA, cysteine protease (3CLpro) with a primary sequence 96% identical to that of SARS-CoV Mpro and whose catalytic site topology is conserved across all betacoronaviruses [14]. The first crystal structure, deposited in February 2020 (PDB ID 6LU7), revealed a homodimeric enzyme in which each 306-residue monomer (Mr = 33.8 kDa) folds into three domains (Fig. 2A) [10, 15]. Domains I (residues 8–101) and II (102–184) form a six-stranded antiparallel β-barrel characteristic of chymotrypsin-like and picornaviral 3C proteases, with the catalytic dyad (His41–Cys145) located at the interface. Domain III (201–303) comprises a cluster of five α-helices arranged in a globular fold and is connected to domain II by a long flexible loop (185–200). This domain plays a crucial role in regulating Mpro dimerization, primarily through a salt bridge formed between Glu290 of one monomer and Arg4 of the opposing monomer [16]. Domain III is unique to viruses of the order Nidovirales, which include coronaviruses, and it is absent in other viral proteases [17].

Although each Mpro monomer contains a complete active site and adopts a native-like tertiary structure, isolated monomers are catalytically inactive [16]. Similarly to the SARS-CoV main protease, this loss of activity is attributed to distortion of the monomeric active-site architecture, specifically occlusion of the S1 subsite, which disrupts productive recognition of the substrate P1 glutamine residue [18]. In the functional homodimer, the two catalytic sites are located at the interfaces between domains I and II of each monomer, and enzymatic activity is achieved only upon dimerization. This activation depends critically on interactions involving the N-terminal 1–7 residues ( N-finger) of one monomer with the Glu166 of the opposing monomer, a contact that is essential for the proper formation and stabilization of the S1 substrate-binding pocket [16].

The active site of Mpro, including its substrate-binding pocket, is highly conserved among different coronaviruses. It is conventionally divided into a series of subsites, denoted S and S′, each of which accommodates a single substrate residue located before (P) or after (P′) the scissile peptide bond. Within the substrate-binding cleft of Mpro, five principal subsites can be identified: S1′, S1, S2, S3, and S4, which interact with the P1′, P1, P2, P3, and P4 positions of the substrate, respectively. The S1, S2, and S4 subsites form well-defined pockets within the protease cavity, whereas the S3 subsite, located closer to the protein surface, exhibits a less well-defined and more solvent-exposed architecture [10, 16, 19].

Substrate recognition is primarily driven by the S1 pocket, defined by the loop spanning Phe140 to Cys145 and by the residues His163, Glu166, and His172, which collectively enforce specificity for a Gln residue at P1 (Fig. 2B) [20]. The adjacent S1′ subsite is shallow and accommodates small residues such as Ser, Gly, or Ala at P1′ [10]. The S2 pocket—formed by His41, Met49, Tyr54, Met165, and Asp187—accommodates hydrophobic residues, typically Leu or Phe, at P2 [21]. The solvent-exposed S3 site tolerates broader variability at P3, while S4 preferentially binds to smaller hydrophobic side chains at P4 and is formed by the side chains of Met165, Leu167, Phe185, Gln189, and Gln192 [10, 16] (Fig. 2B). In summary, substrate specificity of Mpro is primarily dictated by strict structural requirements at the S1, S2, and S4 subsites, which coordinate the highly conserved P1, P2, and P4 substrate residues, respectively. This is followed in importance by the S1’ subsite, whereas the more solvent-exposed S3 and S3’ exhibit broader tolerance for varying substrate residues and contribute mainly to substrate stabilization within the active-site cleft.

SARS-CoV-2 Mpro displays a high degree of specificity for substrates containing the consensus cleavage sequence (Leu)–Gln↓(Ser/Ala/Gly), in which proteolysis occurs immediately C-terminal to the conserved Gln residue. This target amino acid sequence is not recognized by any human proteases, thereby enabling the design of selective Mpro inhibitors [22]. The active site of each Mpro monomer contains a catalytic dyad composed of Cys^145^ and His^41^, located within the S1 subsite substrate-binding cleft between domains I and II. This dyad is considered non-canonical because, unlike classical serine and cysteine proteases, it lacks a stabilizing acidic residue (Asp/Glu) that would otherwise complete a catalytic triad. In this system, like MERS-CoV and SARS-CoV Mpro, the absence of the standard third catalytic element (acidic residues) is compensated by the presence of a buried water molecule, which forms a hydrogen-bonding network involving the His41 residue and the surrounding residues [21, 23, 24].

Mpro is highly conserved among coronaviruses and exhibits a high degree of sequence homology with homologous proteases from other members of the family, such as MERS-CoV and SARS-CoV. Particularly, the SARS-CoV-2 Mpro shares approximately 96% amino acid sequence identity with the SARS-CoV Mpro, and the two enzymes display comparable catalytic efficiencies. Consequently, their enzymatic mechanisms are largely conserved and can be described as proceeding through a series of well-defined steps.

**Fig. 3:**
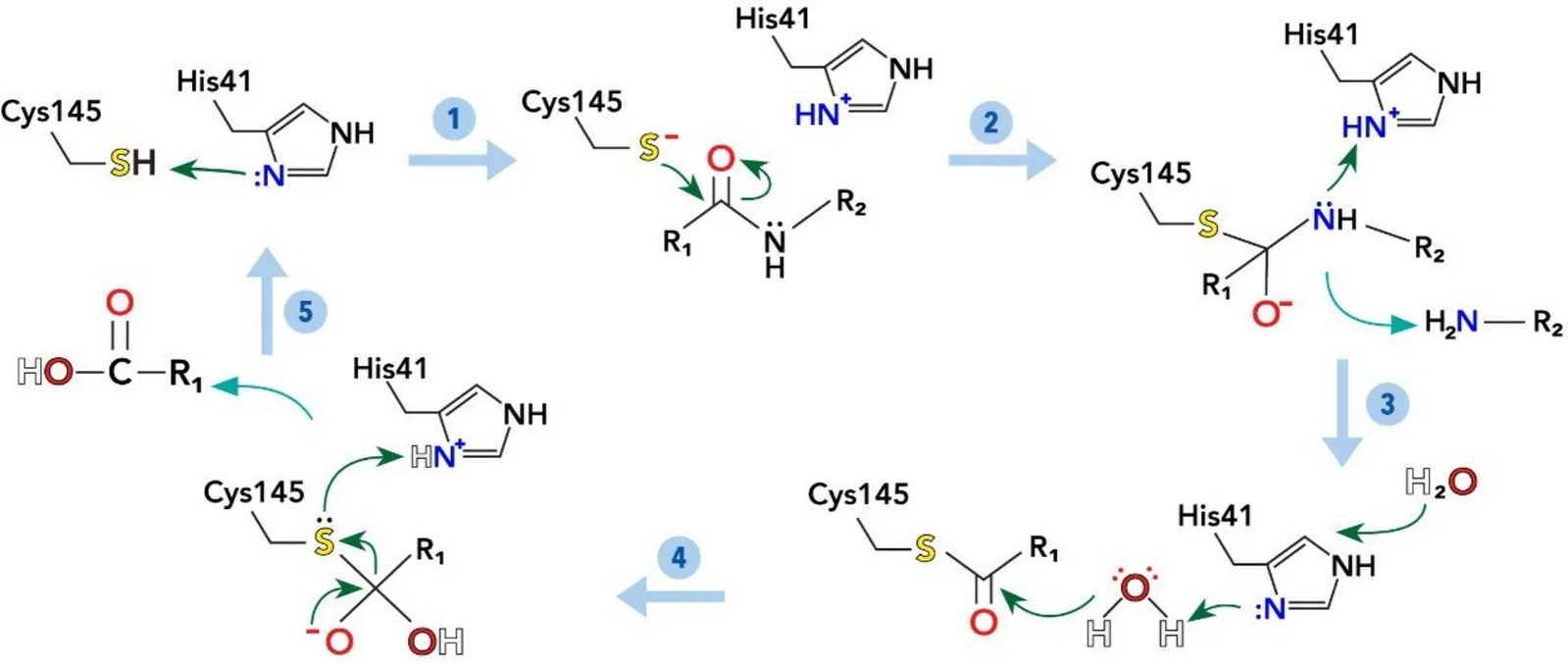
Catalytic mechanism of substrate hydrolysis by SARS-CoV-2 Mpro. **(1)** Cys_145_ thiol deprotonation. **(2)** Nucleophilic attack. **(3)** Release of N-terminus substrate. **(4)** Thioester hydrolysis. **(5)** Release of carboxylic acid and catalytic dyad restoration

In the pre-catalytic resting state, Cys145 predominantly exists in its thiol form (–SH), and His41 remains neutral (Fig. 3). As a result, the dyad is not fully ionized, which helps minimize nonspecific proteolysis. This behavior contrasts with that of canonical cysteine proteases, where a pre-formed thiolate–imidazolium ion pair is commonly observed. In Mpro, the imidazole ring of His41 is only weakly activated by a water molecule. Its protonation state is influenced by substrate binding, local hydrogen-bond networks with Phe140, His163, and Glu166 (including structured water molecules) stabilizing the oxyanion hole [25], and homodimerization, which is essential for enzymatic activity.

In the enzymatic mechanism, Cys145 acts as a nucleophile, while His41 functions as a general base/acid, operating without the strong electrostatic stabilization of an acidic residue. Consequently, His41 is not permanently polarized, rendering catalysis more tightly regulated and context-dependent [26–28]. Upon substrate binding, conformational changes reposition His41, enabling it to abstract a proton from Cys145. The activated Cys145 thiolate then performs a nucleophilic attack on the carbonyl carbon of the scissile peptide bond of glutamine residue, leading to the formation of a tetrahedral oxyanion intermediate, which is stabilized within the oxyanion hole. At the molecular level, the Oβ atom of the P1 glutamine engages the oxyanion hole formed by the Phe140-Cys145 loop within the S1 subsite, thereby correctly positioning the scissile bond. Accordingly, the P1 position almost invariably requires glutamine or a glutamine-mimetic (lactam) warhead, a feature that has been widely exploited in inhibitor design [22]. The resulting charge development is stabilized by the oxyanion hole, formed mainly by the backbone NH groups of Gly143, Ser144, and Cys145, as well as by nearby water molecules. This process generates a transient ion pair, consisting of a Cys145 thiolate (S^−^) and a protonated His41 (HisH^+^). The collapse of this first tetrahedral oxyanion intermediate, accompanied by proton donation from His41 to the leaving amine group, results in the release of the N-terminal product and formation of a thioester acyl-enzyme intermediate [29]. Subsequently, deacylation (hydrolysis) occurs when a water molecule enters the active site. His41 again acts as a general base, activating the water molecule for nucleophilic attack on the thioester carbonyl. This leads to the formation and collapse of a second tetrahedral intermediate, releasing the C-terminal product and restoring Cys145 to its thiol state [28].

This non-canonical catalytic mechanism is advantageous because of the transient activation of the cysteine nucleophile by His, in the absence of a stabilizing acidic residue, strongly links catalysis to substrate positioning, active-site solvation, and conformational communication across the dimer interface. These features confer high substrate specificity, enable allosteric regulation, and create vulnerabilities to inhibitors that disrupt dimerization or His41 dynamics. Thus, the reactivity of the Cys145-His41 dyad is finely modulated by dimerization and long-range intramolecular interactions.

Beyond the active site, enzymatic activity depends critically on the quaternary structure of Mpro. The N-terminal Ser1–Gly7 “finger", disordered in the monomer, docks between domains II and III of the partner protomer within the functional dimer. The NH_2_ of Ser1 forms hydrogen bonds with Glu166 and Phe140 of the opposite chain, positioning Phe140 for π-stacking with His163 and thereby stabilizing the S1 pocket for optimal P1-Gln recognition [30]. Disruption of this interface, by deletion or extension of the N-finger, abolishes enzymatic activity [31], while tags at the C-terminal end impair dimerization and catalytic efficiency [32]. Consequently, tag-free, native termini are essential for accurate biochemical and structural characterization of Mpro.

## Structural basis of oligomerization and allosteric regulation in Mpro

Several studies have shown that SARS-CoV-2 Mpro is active as a homodimer. This is readily seen in the structure, whereas Ser^1^ from one monomer forms hydrogen-bonds with Phe140, Glu166, and His172 of the partner subunit, helping organize the S1 pocket [10, 33]. In recent studies, multiple groups have demonstrated that catalysis is intrinsically asymmetric and depends on long-range allosteric communication (~ 40 Å) between the two active sites. Substrate or inhibitor binding to one protomer can influence the structural dynamics and reactivity of the second, leading to temporary asymmetric conformations within the dimer and, in some contexts, signatures of cooperativity [34, 35].

Cooperativity and functional asymmetry remain an active area of investigation. Minicozzi et al*.* [36] found that the initial substrate binding to one active site allosterically perturbs the partner site by inducing entropic compensation: the occupied monomer becomes more ordered, while the unbound monomer becomes more dynamic and expels water molecules, changes that together facilitate catalytic turnover [36]. Kinetic measurements further support positive kinetic cooperativity—enhanced processing efficiency despite unchanged substrate binding affinity—highlighting that the enzyme’s cooperative behavior is rooted in dynamical and conformational communication rather than improved thermodynamic binding [37, 38]. Structural, mutagenesis and enzymology evidence supports a model in which Mpro maturation and dimerization follows a highly coordinated sequence of events: (1) N-terminal intramolecular cleavage at the NSP4-NSP5 junction, followed by N-finger insertion into the opposing monomer, restructuring the active site and establishing a stable dimer interface; (2) intermolecular C-terminal cleavage, primarily mediated by neighboring monomers; and (3) subsequent formation and release of mature homodimeric Mpro [38, 39].

The dimerization of SARS-CoV-2 main protease (Mpro) has been the subject of several biophysical investigations using a variety of experimental approaches and techniques, including size-exclusion chromatography (SEC) [40] and SEC with multi-angle light scattering (SEC-MALS) [41, 42], sedimentation velocity analytical ultracentrifugation (SV-AUC) [42, 43], microscale thermophoresis (MST) [44], differential scanning fluorimetry (DSF) [42], isothermal titration calorimetry (ITC) [42, 45], mass spectrometry (MS) [41, 46], molecular dynamics simulations (MD) [47], synchrotron small-angle X-ray scattering (SAXS) [16, 44], and structure determination by X-ray diffraction and cryo-electron microscopy [41, 45]. The monomer–dimer dissociation constants (K_D_) estimated by these techniques were quite variable and dependent on methodological differences and experimental conditions, such as protein concentration, ligands, and protein design.

Early SV-AUC studies estimated K_D_ values in the low micromolar range (2.5 µM), indicating that Mpro exists in a dynamic equilibrium between monomeric and dimeric states in solution, but with a significant population of homodimers at physiologically relevant concentrations [43]. Subsequent analyses using native SEC-MALS reported comparable or slightly weaker affinities, with K_D_ values ranging from up to 2 μM, depending on buffer composition and protein concentration [41, 42]. SAXS measurements on SARS-CoV-2 Mpro, in the absence of ligands and combined with biophysical modeling, also support low micromolar homodimer stability, with estimates of ~7 μM and ~5 µM [16, 44]. In contrast, native mass spectrometry-based assays have reported a markedly tighter equilibrium, with a K_D_ of ~0.14 μM, suggesting that the enzyme has a strong intrinsic propensity to dimerize in solution [46].

These studies highlight a range of K_D_ values spanning sub-micromolar to low micromolar concentrations, which likely reflect differences in protein constructs, buffer conditions, temperature, and detection methods. Nonetheless, the preponderance of evidence indicates that Mpro favors the dimeric state in solution, consistent with the requirement that dimerization is necessary for full catalytic activity. Despite the quantitative variability, a consistent conclusion emerges from these studies: Mpro displays a clear thermodynamic preference for the dimeric state, and dimerization is tightly coupled to active-site integrity and enzymatic competence. The observed spread in reported K_D_ values likely reflects the sensitivity of the monomer–dimer equilibrium to N-terminal processing, conformational dynamics, solution conditions, and allosteric effects, rather than fundamental disagreement regarding the oligomeric behavior of the enzyme. Furthermore, the addition of citrate and other kosmotropic agents to the assay buffer can promote Mpro dimerization at sub-K_D_ concentrations, maintaining or even enhancing enzymatic activity by stabilizing the active dimer and providing a workaround for highly sensitive enzymatic assays [34].

## Strategies for the tag-free heterologous production of Mpro

Recombinant SARS-CoV-2 Mpro is routinely produced in *Escherichia coli* with the aid of affinity/solubility tags. Hexa-histidine (6×His), small ubiquitin-like modifier (SUMO), glutathione *S*-transferase (GST) and maltose-binding protein (MBP) tags are inserted at either the N- or C-terminus, invariably followed by a protease-cleavable linker that allows tag removal after purification (Table 1). Most constructs are expressed in BL21(DE3)-derived strains. Self-processing linkers that reproduce the viral N-terminal cleavage site (SAVLQ↓) are frequently engineered between the *tag* and the protease. During expression, Mpro removes its own N-terminal tag, thereby ensuring correct folding and dimerization, while the affinity tag remains attached to the C-terminus [48].

**Table 1:** Tag-free heterologous production strategies for Mpro. Schematic representation of the fusion constructs used for tag-free Mpro production. The SARS-CoV-2 main protease (Mpro) is expressed as a fusion protein flanked by affinity tags to facilitate purification and subsequent tag removal

Expression construct	Plasmid and cell strains	Expression broths, temperature and induction	Purification strategies				Reference
			1º Step	2º Step	3º Step	4º Step	
1	pGEX-6P-1, *E. coli* BL21 (DE3) pLysS	LB, 0.5 mM IPTG (3 h at 37 °C followed by 10 h at 16 ºC)	**AF **(Ni–NTA), followed by tag removal by HRV 3C-GST	**AF** (GSTrap FF)	**SEC** (Superdex 75 10/300)		[16]
2	pGEX-6P-1*, E. coli* BL21 (DE3)	LB, 1 mM IPTG (5 h at 16 °C)	**AF** (HisPur Ni–NTA), followed by tag removal by HRV 3C-6xHis	**AF** (HisPur Ni–NTA)	**SEC** (Sephacryl S-200 HR 16/60)		[35]
3	pET11a, *E. coli* BL21 (DE3)	LB, 1 mM IPTG (3 h at 37 °C)	**AF** (His-Trap), followed by tag removal by Factor Xa	**IE** (HiTrap Q FF)			[49]
4	pET11a, *E. coli* BL21 (DE3)	LB, 12.5 mL 8% lactose, 5 mL 60% glycerol, 2.5 mL 10% glucose (final volume of 500 mL)	**IE** (Q-Sepharose Fast Flow)	**HIC** (Phenyl Sepharose 6 Fast Flow Hi Sub)	**IE** (Mono S)	**SEC** (HiLoad 26/60 Superdex 200)	[50]
5	pET15b, *E. coli* BL21 (DE3)	TB, 1 mM IPTG (16 h at 16 °C)	**AF** (HisTrap FF), followed by tag removal by thrombin	**AF** (HisTrap FF)	**SEC** (HiLoad 16/60 Superdex 200)		[51]
6	pGEX-4 T-1*, E. coli* Rosetta 2 (DE3) pLysS	LB, 1 mM IPTG (5 h at 25 ºC)	**AF** (Glutathione Sepharose 4 Fast Flow), followed by tag removal by Factor Xa	**AF** (Glutathione Sepharose 4 Fast Flow)			[52]
7	pET28a, *E. coli* BL21 (DE3)	2xYT, 1 mM IPTG (3 h at 37 °C)	**AF** (Ni-chelating column) (GenScript), followed by tag removal by SENP	**IE** (Q Sepharose)	**SEC** (Superdex 200)		[53]
8	pD451-SR, *E. coli* BL21 (DE3)	LB, 0,2 mM IPTG (18 h at 18 °C)	**AF** (HisTrap FF), followed by tag removal by HRV 3C-6xHis	**AF** (HisTrap FF)			[54]
9	pGEX-4 T-1, *E. coli* BL21 (DE3)	LB, 0,5 mM IPTG (16 h at 16 °C)	**AF** (Ni–NTA), followed by tag removal by HRV 3C-GST	**AF** (GST affinity column)	**AF** (Ni–NTA)	**IE** (HiTrap Q)	[40]
10	pGEX-6P-1, *E. coli* BL21-Gold (DE3)	LB, 0,5 mM IPTG (5 h at 37 °C)	**AF** (HisTrap FF), followed by tag removal by HRV 3C-GST	**AF** (GSTrap FF)			[55]
11	pGEX-6P-1, *E. coli* BL21 (DE3)	YT, 0,5 mM IPTG (5 h at 37 °C)	**AF** (HisTrap FF), followed by tag removal by HRV 3C-GST	**AF** (GSTrap FF and HisTrap FF connected in tandem)	**SEC** (HiLoadTM 16/600 SuperdexTM 200 pg)		[56]

AF, affinity chromatography; IE Ion-exchange chromatography; SEC, size-exclusion chromatography; HIC, hydrophobic interaction chromatography; Ni–NTA, Nickel–nitrilotriacetic acid affinity resin; GSTrap FF, Glutathione Sepharose Fast Flow affinity column; HisTrap FF, Nickel affinity column for polyhistidine-tagged proteins; Q Sepharose, quaternary ammonium groups sepharose Strong anion-exchange resin and fast flow (HiTrap Q FF); Mono S, MonoBeads Tricorn column polystyrene/divinyl benzene strong cation-exchange column; Superdex/Sephacryl, Size-exclusion chromatography resins; Phenyl Sepharose, Hydrophobic interaction chromatography resin; HRV 3C, Human rhinovirus 3C protease; FXa, Factor Xa protease; SENP, SUMO-specific protease; Thro, thrombin; LB, Luria–Bertani medium; TB, Terrific Broth medium; YT/2xYT, Yeast extract–tryptone medium double strength; IPTG, Isopropyl β-D-1-thiogalactopyranoside; GST, Glutathione S-transferase affinity tag; 6xHis, Hexahistidine affinity tag. “1º Step”, “2º Step”, etc. refer to the sequential purification steps applied after cell lysis.

The strategies compiled for recombinant production of SARS-CoV-2 Mpro (Table 1) show a high degree of convergence in expression host, induction conditions, and purification workflows, with variations mainly related to affinity tags and polishing steps. Most studies employ *E. coli* expression systems based on the lactose operon (lac) from pET (strong bacteriophage T7 promoter and His tags) and pGEX (tac promoter and GST fusion tag) vector series (Table 1). In general, pET vectors are preferred for achieving high expression yields, whereas pGEX vectors are often more effective for producing soluble and stable protein.

The expression host strains used were E. coli BL21 (DE3)-derived strains, reflecting the enzyme’s cytosolic nature, lack of complex post-translational modifications, and compatibility with high-yield bacterial systems (Table 1). Culture media commonly include LB, TB, or 2×YT, with protein expression typically induced using IPTG at concentrations ranging from 0.2 to 1 mM, or 8% lactose. A frequent strategy to enhance solubility and proper folding is temperature downshifting after induction, with many protocols using 16–18 °C for extended expression periods (Table 1).

Regarding construction design, fusion tags play a central role in enabling efficient purification and improving solubility. The most widely used tags are polyhistidine (6xHis) and glutathione S-transferase (GST), introduced via plasmids such as pET, pGEX, and related derivatives. In several strategies, Mpro is flanked by removable affinity tags separated by specific protease cleavage sites, most commonly HRV 3C protease, but also thrombin, Factor Xa, or cysteine sentrin-specific protease (SENP) that regulate SUMOylation (Table 1). This design allows affinity capture followed by enzymatic tag removal to obtain tag-free enzymes suitable for structural and kinetic studies. Additional amino acid residues at either terminus, particularly the N-terminus, impair Mpro dimerization and decrease catalytic efficiency [40]. Consequently, constructs yielding tag-free Mpro are preferred for enzymatic and structural studies. Among these, designs containing a N-terminal self-cleavage sequence mimic Mpro maturation process, as seen in constructs (2), (4), (9), and (11). This process ensures that the N-terminus begins with the native Ser1, which is essential for proper dimerization and catalytic activity.

Constructs that exploit the partially overlapping substrate recognition of Mpro and HRV 3C protease are elegant strategies to obtain authentic termini. For instance, Lee J. et. al [35] employed a design in which Mpro is flanked N-terminally by a segment reproducing the natural NSP4/NSP5 junction (SAVLQ↓SG) and C-terminally by a hybrid cleavage site followed by a 6xHis tag. The C-terminal site is engineered asymmetrically: the non-prime portion, which remains part of the mature enzyme, is left unmutated so that cleavage regenerates the authentic C-terminal Gln306, whereas the prime-side residues are replaced by the Gly-Pro motif, designed exclusively for HRV 3C recognition [35]. This strategy was originally developed for the homologous SARS-CoV Mpro and became the gold standard route to mature, tag-free Mpro production [57]. Modifications to the labile self-processing sites between the Mpro sequence and purification tags have also been used to slow down cleavage. This delay enables effective binding to affinity columns, followed by gradual on-column autocatalysis, yielding tag-free enzyme upon elution and circumventing the need for imidazole elution or external protease removal [58]. Purification workflows generally begin with affinity chromatography (AF), most often Ni^2+^-NTA or GST-based resins, which provide the primary capture step. After tag cleavage, in combination with dialyses, a second affinity step is frequently used to remove the cleaved tag and protease, improving sample homogeneity. Ion-exchange chromatography (IEX), using quaternary ammonium groups (Mono Q) or methyl sulfonate groups (Mono S) columns, is commonly incorporated as an intermediate polishing step to separate different oligomeric or contaminant species. Hydrophobic interaction chromatography (HIC) appears less frequently but can be used for additional refinement. Nearly all protocols include size-exclusion chromatography (SEC), typically on Superdex 75 or Superdex 200 resins, to ensure monodispersity, remove aggregates, and obtain the correct dimeric form of Mpro (Table 1).

Overall, the table highlights a broadly consistent production pipeline: bacterial overexpression at reduced temperature, affinity-based capture, proteolytic tag removal, and final polishing by ion exchange and SEC. Differences among protocols mainly reflect optimization choices aimed at maximizing solubility, ensuring complete tag removal, and achieving the high purity and homogeneity required for biochemical, biophysical, and structural characterization of Mpro.

## Methods and strategies for assessing enzymatic activity

Enzyme activity of SARS-CoV-2 Mpro is conventionally assayed using fluorogenic substrates containing a quenched donor–acceptor pair. In these synthetic substrates, a fluorophore and a quencher moiety flank the Mpro cleavage sequence (Table 2). In the intact substrate, proximity-dependent quenching, typically through resonance energy transfer (FRET) or related mechanisms suppresses fluorophore emission. Proteolytic cleavage separates the fluorophore from the quencher, resulting in an increased fluorescent signal that allows continuous kinetic monitoring of Mpro activity. The consensus peptide sequence is derived from the NSP4↓NSP5 autoprocessing site (TSAVLQ↓SGFRKM) that displays the highest catalytic efficiency for Mpro [59]. Shorter or solubility-enhanced variants are often generated to reduce synthesis cost, susceptibility to interference, false positives and improve the hydrolysis rate [60, 61]. Typical donor–quencher pairs are EDANS–DABCYL, MCA–DNP and Abz–Y(3-NO_2_) (Table 2).

**Table 2:**
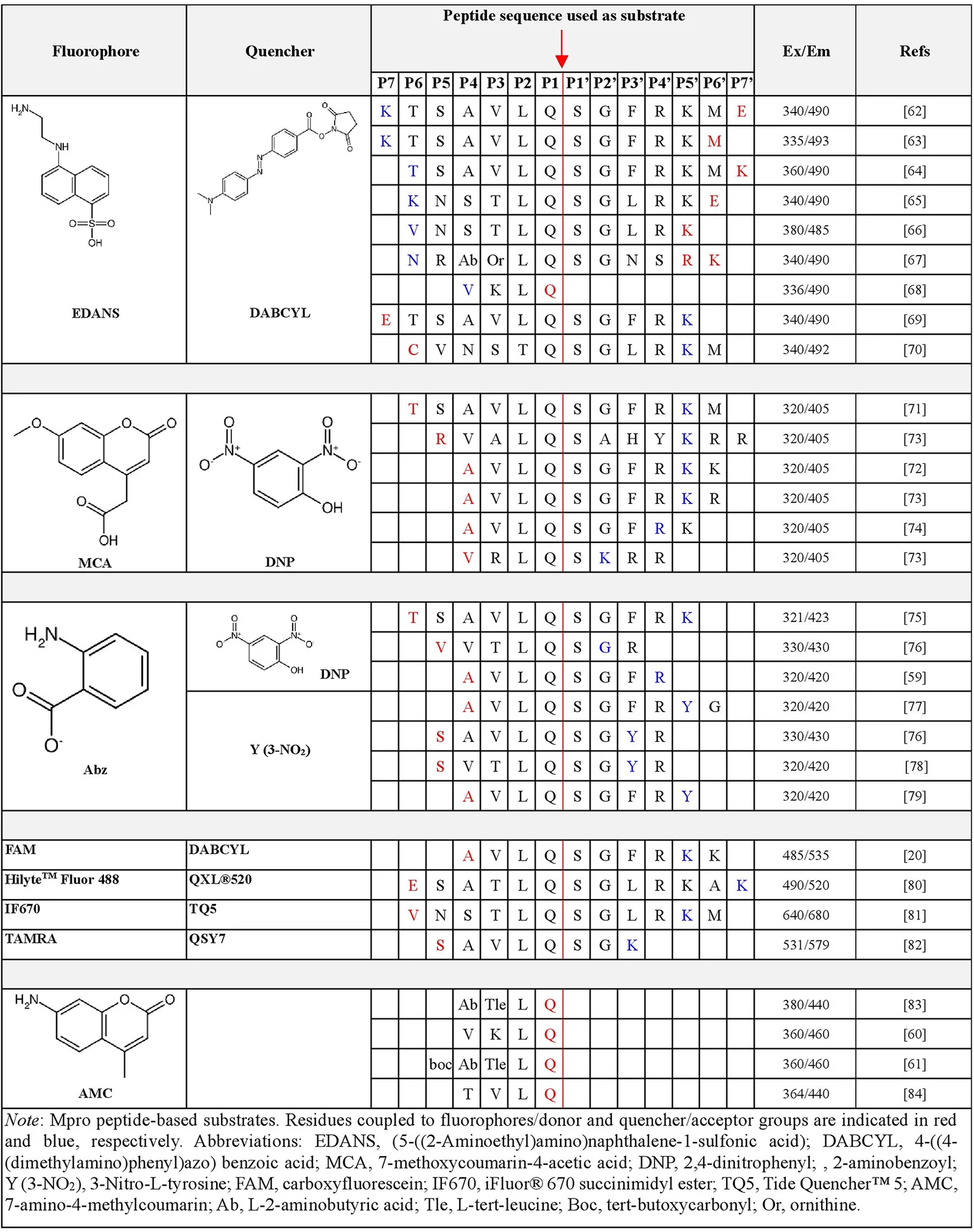
Design strategies of peptide-based substrates for assessing Mpro activity [20, 59–84]

In summary, the table reveals that fluorescence-based peptide substrates are the dominant tools for measuring SARS-CoV-2 Mpro activity, with most assays relying on Förster resonance energy transfer (FRET) pairs flanking short peptides derived from native viral cleavage sites. The most widely used donor–acceptor combination is EDANS/DABCYL, typically monitored at excitation/emission wavelengths around 340/490 nm. Alternative FRET systems such as MCA/DNP and Abz/DNP are also frequently employed, offering different spectral windows that can reduce interference from test compounds or buffer components. More recent studies have introduced longer-wavelength fluorophore-quencher pairs (e.g., FAM/DABCYL, Hilyte Fluor 488/QXL520, and IF670/TQ5) (Table 2), which improve signal-to-noise ratios and are particularly advantageous for high-throughput screening due to reduced background fluorescence.

Across studies (Table 2), substrate sequences show strong conservation around the canonical Mpro cleavage motif, with the core P4-P1 residues most represented as A/V-V/L-L-Q↓S, where cleavage occurs between the P1 glutamine and the P1′ serine. Variations are mainly introduced at flanking positions (P5-P7 and P2′-P7′) to optimize solubility, fluorescence efficiency, or enzyme kinetics without disrupting recognition. The strict conservation of glutamine at the P1 position and small residues such as serine, glycine, or alanine at P1′ reflects the well-established substrate specificity of Mpro. Substrate design has also been driven by systematic specificity profiling. Hybrid Combinatorial Substrate Libraries (HyCoSuL), which systematically probe individual substrate positions using natural and non-natural amino acids, revealed that selected non-proteinogenic residues at P4–P2 can improve Mpro substrate recognition and cleavage efficiency, while confirming its strong preference for Gln at P1. This profiling led to the development of the optimized fluorogenic substrate Ac-Abu-Tle-Leu-Gln-ACC [85], whose Abu–Tle–Leu–Gln recognition motif is also present in AMC-based substrates listed in Table 2 [61, 83]. Complementary high-resolution substrate-specificity profiling and proteomics-based characterization of P6–P6′ cleavage-site preferences have further refined our understanding of Mpro substrate recognition and guided the optimization of fluorogenic substrates included in Table 2 [59, 73].

Methodologically, most assays employ internally quenched substrates that produce an increase in fluorescence upon proteolytic cleavage, enabling continuous kinetic measurements. This real-time format supports accurate determination of kinetic parameters (K_m_, k_cat_) and inhibitor potency (IC_50_, Ki). AMC-based substrates represent a simpler alternative, in which fluorescence is released after cleavage of a short peptide-AMC conjugate (Table 2); however, these are less common and typically used in high-throughput inhibitor screening assays. In general, despite diversity in fluorophores and minor sequence modifications, the field converges on a shared design principle: short peptides mimicking natural cleavage sites, labeled with compatible FRET pairs, enabling sensitive, continuous, and high-throughput quantification of Mpro enzymatic activity. Differences among methodologies mainly reflect optimization for detection wavelength, assay robustness, improved solubility, and compatibility with inhibitor screening platforms. For more details regarding the suitability of Mpro substrates for specific assay applications, we recommend the excellent work of Voget and Gütschow [86]. Activity-based probes (ABPs) provide complementary tools for investigating Mpro activity. These probes typically combine a peptide recognition element, an electrophilic group that covalently reacts with the catalytic cysteine, and a detectable reporter or affinity tag. Such probes have been developed to label and visualize active Mpro in patient-derived samples [85]. Unlike fluorogenic substrates, which report catalytic turnover and support determination of kinetic parameters and inhibitor potency, ABPs primarily detect protease populations with accessible and reactive active sites. They can therefore support studies of target engagement and, under appropriate labeling and calibration conditions, quantification of active enzyme populations through active-site titration [85, 87].

The substantial cost associated with large-scale chemical synthesis of FRET or chromophore-based substrate peptides often limits their applicability in high-throughput inhibitor screening (HTS) campaigns. To bypass this limitation, reporter-based assays have been developed in which Mpro-cleavable sequences are inserted between two fluorescent or bioluminescent proteins. These constructs can be deployed either in cell-based assays, in which plasmids encoding the reporter and Mpro are co-transfected and intracellular FRET is monitored, or conventional *in-vitro* biochemical assays using purified fusion protein obtained by heterologous expression [88]. These methods employ various biological mechanisms for readout, often based on fluorescent/luminescent proteins to create gain or loss-of-signal assays. Examples include, luciferase [89–92], cyan and yellow fluorescent proteins (CFP-YFP) pairs and their optimized variants [93–95], mCherry-GFP systems [67, 96], as well as alternative configurations such as biotin-avidin systems (BAS) [32], phycobiliprotein-based reporter assay [97] and dimerization-dependent red fluorescent protein (ddRFP) [98]. Alternative methods for inhibitor screening and Mpro activity detection that rely on electrochemical signaling have also been developed [99, 100]. These approaches are based on a scissile peptide sequence inserted between an electrode and a redox reporter. Mpro-mediated hydrolysis leads to the diffusion of the reporter away from the surface, resulting in a measurable decrease in the voltametric current. This ‘signal-off’ mechanism provides a highly sensitive readout for quantifying enzymatic activity and evaluating the efficacy of potential inhibitors.

## Mpro kinetic parameters and assay conditions

A significant degree of variability has been observed across reported kinetic parameters for Mpro, as summarized in Table 3. A lack of consensus on Mpro kinetic behavior compromises the reliability, comparability of results and conclusions drawn from experiments, especially in the screening of the inhibitory capabilities of new compounds. Ideally, the assessment of inhibitors should be done with the fully active enzyme in its dimeric form. The wide range of reported K_m_ and k_cat_ values, even for the same substrate, may be attributed to several factors, such as: (i) poor substrate solubility [60, 68]; (ii) fluorescence quenching by the inner-filter effect of cleaved product molecules or neighboring substrates; (iii) variability of assay conditions (temperature, pH, buffer composition, etc.); (iv) varying monomer–dimer ratios; (v) different levels of enzyme purity and structural integrity due to variations in purification protocols; (vi) differences in the kinetic model and fitting algorithm chosen (e.g. Michaelis–Menten vs. cooperativity kinetic models, such as the Hill equation). To circumvent the methodological artifacts and limitations discussed above, several groups have proposed and implemented standardization strategies and alternative methods to assay enzyme activity. Specifically, Voget et al. [87] developed an active-site titration method in which a fluorophore-labelled, covalent and irreversible inhibitor is used to quantify the concentration of catalytically competent Mpro, thereby excluding contributions from precipitated, misfolded or fragmented enzyme [87]. The application of this corrected enzyme concentration resulted in the convergence of kinetic parameters for a panel of commonly employed FRET-based substrates to a narrow, consistent range. Following the same principle of considering only the most active enzyme population for kinetic characterization, Chen et al. [101] determined the composition ratio of monomers and dimers and corrected the enzyme concentration to reflect only the activity of the dimeric, and most active form of Mpro [101].

**Table 3:** Enzymatic assay conditions and tag-free Mpro kinetic parameters

Substrate	Enzymatic assay buffer and temperature	[Mpro] (nM)	K_m_ (µM)	V_max_ (nM/s)	k_cat_ (min^−1^)	k_cat_/K_m_ (s^−1^ M^−1^)	Reference
Dabcyl-KTSAVLQSGFRKME-Edans	20 mM Tris–HCl (pH 7,5), 100 mM NaCl, 5 mM DTT; RT	5000	110.3	7.076	0.0849	12.69	[104]
	20 mM HEPES (pH 6.5), 120 mM NaCl, 0.4 mM EDTA, 4 mM DTT, and 20% glycerol; 30 °C	100	35.36 ± 2.41	38.97 ± 0.91	23.4	11,000	[2]
	20 mM Tris–HCl (pH 7.3), 100 mM NaCl, 1 mM EDTA, 1 mM DTT; 30 °C	200	43.02 ± 3.23	33 ± 1.83	19.74 ± 1	7666 ± 166.6	[105]
	20 mM Bis (pH 7,0); 25 °C	35	10 ± 3.2		132 ± 18		[106]
	20 mM Tris–HCl (pH 7.5), 1 mM EDTA, 10 mM DTT, and 0.01% BSA; n.d	3	14.92		42	46,825	[62]
	Tris–HCl (pH 7.3), 100 nM NaCl, 1 mM EDTA, 5 mM Tris (2-carboxyethyl) phosphine; n.d	30	26.4 ± 9.8		45.6 ± 24.6	31,500 ± 18,600	[107]
	20 mM Tris pH 8.0, 150 mM NaCl, 1 mM TCEP, and 3% DMSO	150	16.5 ± 1.6		3.12 ± 0.18		[108]
Hilyte™Fluor488-ESATLQSGLRKAK-QXL®520	50 mM Tris HCl (pH 7), 150 mM NaCl, and 0.1 mg/mL BSA; 37 °C	20	19.13	87.7 RFU/s		7000	[80]
Abz-AVLQ-COOH	50 mM Tris–HCl (pH 7.3), 1 mM EDTA, 1 mM DTT; 30 °C	10	26.0 ± 1.3	16.83 ± 2	102 ± 12		[79]
MCA-AVLQSGFR-Lys(Dnp)-Lys-NH_2_	50 mM Tris–HCl (pH 7.3), 1 mM EDTA; n.d	30	33.12	50		7608	[109]
	20 mM Tris–HCl (pH 7.5), 300 mM NaCl, 1 mM TCEP; 30 °C	500	3.93 ± 0.46		4.44 ± 0.144	19,000 ± 3200	[110]
	50 mM Tris–HCl (pH 7.3), 1 mM EDTA; 30 °C	200				28,500	[10]
	50 mM Tris–HCl, 1 mM EDTA; RT	100	1.10 ± 0.22				[111]
	20 mM Tris (pH 6.5), 0.4 mM EDTA, 120 mM NaCl, 20% glycerol; n.d	100	39.1 ± 0.8	106.3 ± 6		27,186	[112]
	20 mM Tris–HCl (pH 7.5), 300 mM NaCl, 1 mM TCEP; 30 °C	500	5.31 ± 0.44		6.78 ± 0.09	21,300 ± 2200	[113]
	50 mM Tris–HCl (pH 7.5), 1 mM EDTA; n.d	200	5.4 ± 4.13	0.68 ± 0.08		6296	[114]
	50 mM Tris–HCl (pH 7.5), 1 mM EDTA; n.d	200	7.22 ± 2.48	1 ± 0.08		6925	[115]
	10 mM sodium phosphate (pH 7.4), 150 mM NaCl, 1 mM EDTA, 1 mM DTT; 37 °C	1500	70.75 ± 3.6			13,062	[116]
	50 mM HEPES (pH 7.5) 0.1 mg/mL BSA, and 0.01% Triton X-100; 37 °C	200	8.9 ± 0.8		1.44 ± 0.21	31,785	[101]
	50 mM Tris·HCl (pH 7.3), 1 mM EDTA; 30 °C	96				25,600	[117]
	20 mM Bis–Tris (pH 7.0); RT	80				14,190 ± 420	[60]

EDANS, (5-((2-Aminoethyl)amino)naphthalene−1-sulfonic acid); DABCYL, 4-((4-(dimethylamino)phenyl)azo) benzoic acid; MCA, 7-methoxycoumarin-4-acetic acid; DNP, 2,4-dinitrophenyl; Abz, 2-aminobenzoyl; DTT, dithiothreitol.

The inner-filter effect is a phenomenon in which the measured fluorescence signal is artificially reduced due to the emission light absorption by surrounding molecules in the sample, such as the uncleaved substrate or cleaved product, so that only a fraction of the fluoresced light reaches the detector. The magnitude of this effect is highly dependent on the sample pathlength and concentration of quenching components and can lead to an observed non-linearity between fluorescence and analyte concentration at higher substrate concentrations. The inner-filter effect associated with FRET-based substrates can sometimes interfere with the measurement of the kinetic parameters, since it can lead to lower fluorescence detected and consequently lower K_m_ and V_max_ values [102]. Inner-filter correction methods can be applied to address this issue and enable more accurate kinetic parameters determinations. For instance, calibration curves measuring the fluorescence of the free fluorophore across varying concentrations can be used in the correction of the initial velocities measured at substrate concentrations affected by the inner-filter effect [102]. Examples of the effective application of this corrective strategy in the context of Mpro activity assays include the work by Lee et al. and Quan et al. [35, 103]. While non-FRET-based kinetic assays can mitigate the optical artifacts and solubility issues common to fluorogenic substrates [60, 68, 98], they often introduce new limitations. Techniques such as LC–MS, although more reliable, are often labor-intensive and costly [40].

Because kinetic parameters are heavily dependent on experimental conditions, reported values for the same substrate can differ considerably. Consequently, rigorous comparisons among substrates require identical assay conditions, ideally performed within the same laboratory and experimental setup. Beyond the variability introduced by experimental conditions and inner-filter effects, the MCA-Dnp substrates generally exhibit lower K_m_ values than other FRET pairs. However, this higher apparent affinity may also be attributed to differences in the specific peptide sequences. Overall, longer peptide moieties containing the MCA-Dnp and Dabcyl-Edans pairs provide more extensive interactions within the active site, thereby enhancing Mpro recognition, cleavage, and catalytic efficiency (k_cat_/K_m_). Although shorter, non-FRET substrates are cost-effective and easily synthesizable, their use is mostly applied in high-throughput screening (HTS) assays. The Mpro concentrations used in enzymatic characterization assays range from 3 nM to 5 µM (Table 3). However, most of the studies used concentrations at or under 200 nM. Assays that evaluate Mpro concentration plotted against the hydrolysis rate often show an exponential growth trend in the series of concentrations under 200 nM, followed by a linear series at increasing concentrations. The authors suggest that this behavior is due to the formation of the catalytically competent homodimer at around 100–200 nM enzyme concentration [76, 101].

The activity of Mpro is strongly dependent on the enzymatic assay conditions, including the choice of buffering agent and pH, salt concentration, the presence of organic solvents such as dimethyl sulfoxide (DMSO) and reducing agents like dithiothreitol (DTT) and tris(2-carboxyethyl)phosphine (TCEP). Tris–HCl was the most frequently selected buffering agent (10–50 mM) for determining Mpro kinetic parameters, followed by HEPES and sodium phosphate (Table 3). The pH values used ranged from 6.5 to 8.0, with most studies employing pH 7.3 or 7.5, which is consistent with the optimal pH range for Mpro activity [118]. Optimization experiments evaluating Mpro activity for the AKLQ–AMC substrate showed a strong preference for NaPO_4_ (20 mM at pH 7.0) over Bis–Tris, HEPES, and Tris in the presence of 150 mM NaCl [60]. The highest enzyme activity was observed for 20 mM NaPO_4_ without NaCl. Similarly, Dražić et al. [76, 115] determined the optimal assay conditions for Mpro activity. Their evaluation, utilizing the substrate Abz-VVTLQSG-Dap(Dnp)-Arg-OH, identified the highest Mpro activity under the combination of 300 nM enzyme, 50 mM Tris–HCl, pH 7.4, 100 mM NaCl, 20% ethylene glycol, and 0.0016% of detergent Brij 58. Notably, these optimal assay conditions were determined for the substrates evaluated and should not be extrapolated as the optimal conditions for every Mpro activity assay. Substrate solubility is paramount and should always be considered.

Furthermore, the enzymatic activity of Mpro is strongly regulated by the redox state of the environment. The Mpro sequence contains 12 cysteines, including the conserved catalytic residue Cys_145_, whose oxidation decreases catalytic efficiency, indicating that a reducing state is required to preserve maximal enzymatic efficiency [10, 43]. In addition, Mpro function depends on its homodimeric state, which represents the catalytically active form. Consequently, variations in redox conditions exert a direct influence on the structure, enzymatic activity, and oligomeric state of Mpro. For example, cysteine oxidation in the catalytic site and near the dimer interface has been reported to impair dimerization. In vitro studies have demonstrated a redox-switch regulatory mechanism in Mpro, in which oxidizing conditions promote disulfide bond formation between Cys145 and Cys117. This disulfide–dithiol switch induces structural changes that shift the oligomeric equilibrium from the enzymatically active dimer toward the inactive monomeric state [119, 120]. However, specific cysteine modifications of Mpro—such as the Cys145-Cys117 disulfide bond and Cys300 glutathionylation—are reversible upon reductive treatment and can restore both dimerization and catalytic function [120, 121].

Therefore, activity assays commonly include reducing agents such as DTT or TCEP in the reaction buffer to maintain cysteine residues in their reduced state (Table 3), thereby supporting structural stability and assay reproducibility [122–124]. Several biochemical studies of Mpro have reported a substantial enhancement in enzymatic activity in the presence of DTT at concentrations ranging from 1 to 5 mM [104, 124–126]. As a thiol-based reducing agent, DTT prevents oxidation and disulfide bond formation, thereby preserving the active dimeric form of the enzyme. However, DTT contains free thiol groups; it can interfere with the evaluation of covalent or electrophilic inhibitors by competing with the catalytic cysteine or quenching reactive functional groups, which may lead to an underestimation of inhibitory potency [121–126]. In such cases, non-thiol reducing agents such as TCEP represent an alternative [126].

In contrast, TCEP is a phosphine-based reducing agent that lacks free thiol groups and thereby reduces direct competition with cysteine-reactive compounds. TCEP is functional over a broad pH range and exhibits higher chemical stability in aqueous solutions [124, 126]. In the context of Mpro enzymatic buffer assays, replacing DTT with TCEP can be advantageous for evaluating covalent or electrophilic inhibitors, as it minimizes thiol-mediated quenching reactions and reduces the likelihood of false-negative or the underestimation of inhibition results [127]. Therefore, TCEP is often preferred over DTT in structural and biochemical inhibition studies involving cysteine-targeting compounds, as it reduces the identification of false-positive inhibitors in screening assays and minimizes redox-dependent assay interference [76, 123, 126].

Conflicting evidence exists regarding the effects of DMSO on Mpro activity. Substrate solubility often requires the use of DMSO, and its concentration must be carefully optimized, as excessive levels can reduce enzyme activity. Legare et al. [60] found a negative effect of DMSO at concentrations under 10% on Mpro activity for the AKLQ–AMC substrate in 20 mM NaPO4 (pH 7.0) and 150 mM NaCl [60]. Nguyen et al. [118] reported that at DMSO concentrations below 10% Mpro activity in 50 mM Tris–HCl (pH 7.5) for Dabcyl-KTSAVLQ↓SGFRKME-Edans substrate remained unchanged, but at concentrations above 10% its activity decreased [118]. For the same substrate in 20 mM HEPES (pH 7.0), 150 mM NaCl, 1 mM EDTA, and 1 mM TCEP, Ferreira et al. [128] reported that the catalytic efficiency increased at DMSO concentrations above 10% (up to 20%), possibly due to an improvement in the substrate stability and solubility. This effect was attributed to improved substrate stability and solubility under higher DMSO conditions [128].

## Concluding remarks and recommendations for Mpro biochemical assays

The enzymatic assays for SARS-CoV-2 Mpro reveal pronounced sensitivity to experimental conditions, contributing to variability in reported kinetic parameters across studies [60, 68]. Buffer composition, pH (typically optimal at 7.3–7.5) [60, 76, 118], ionic strength (often 100–300 mM NaCl), and temperature (30 °C or 37 °C preferred over ambient for physiological relevance) require substrate-specific optimization, with all parameters carefully documented to support meaningful comparisons. Mpro functions exclusively as a homodimer, with dissociation constants ranging from 0.14 to 7 µM [16, 42–44, 46]; enzyme concentrations below 100–200 nM commonly results in rate-limiting dimerization and non-linear kinetics [76], whereas ≥100–200 nM concentrations favor the active dimeric state. Active-site titration using fluorophore-labeled irreversible inhibitors provides a reliable means to quantify catalytically competent enzyme, minimizing distortions from misfolded or aggregated material [87, 101].

Construct design is critical: native N- and C-termini are essential for dimerization and catalytic competence, as the N-terminal “finger” (Ser1–Gly7) stabilizes the S1 pocket through specific interactions [16, 129]; any residual affinity tags or extensions impair activity and must be fully removed (e.g., via HRV 3C, thrombin, or SUMO protease) [48], preferably confirmed by mass spectrometry (MS). Redox control is equally important given the enzyme’s 12 cysteines, including the catalytic Cys145 [10, 43]; oxidative inactivation or disulfide formation disrupts dimerization and is best prevented with TCEP (1 mM) rather than DTT when screening covalent or electrophilic inhibitors [121, 122, 124, 126], as DTT’s thiols can compete or quench reactive warheads and produce false negatives [122, 123].

FRET-based substrates remain the standard for continuous monitoring, with the consensus NSP4↓NSP5 sequence (TSAVLQ↓SGFRKM) offering the highest efficiency, though shorter variants improve solubility at the cost of some performance [60, 68]. DMSO effects vary by substrate-buffer pair and should be minimized (typically ≤5%) while confirming solubility and documenting concentrations [60, 118, 128]. Inner filter effect corrections are routinely necessary at higher substrate levels to avoid systematic underestimation of V_max_ and K_m_ [35, 102, 103]. Continuous FRET assays suit detailed mechanistic work, whereas reporter-based or endpoint formats better accommodate large-scale screening, provided hits receive orthogonal confirmation [88, 90–92].

Kinetic analysis benefits from inspecting progress curves for cooperativity or substrate inhibition and applying appropriate models (e.g., Hill equation when warranted) over broad substrate ranges. Finally, reliance on single-assay formats risks undetected artifacts; integration of orthogonal readouts (LC–MS, native MS, SPR, ITC) strengthens conclusions [42, 45, 46], while exhaustive reporting of substrate sequence, enzyme details, buffer conditions, temperature, additives, reducing agents, DMSO content, kinetic model, and replicate statistics enables cross-study comparison and reproducibility. Adoption of these practices—active-site titration, dimer-equilibrium correction, TCEP use for covalent screening, inner filter correction, transparent documentation, and multi-method validation—substantially improves consistency and accelerates reliable identification of Mpro-targeted antivirals.

To synthesize our findings into practical laboratory guidance, we outlined a best practice checklist for future Mpro enzymatic assays. Regarding substrate selection, fluorescent-based designs containing FRET pairs or single fluorophores are preferable. When selecting a FRET pair or a single fluorophore, several critical points should be considered: (i) Spectral overlap: Excitation and emission maxima must not overlap with the optical properties of tested compounds (Table 2 details fluorophore and quencher groups with recommended excitation and emission wavelengths covering a broad spectrum); (ii) Photostability: Photobleaching should be minimized by storing substrate stock solutions in amber glass or dark amber polypropylene tubes; (iii) Solubility and storage: As most organic fluorophores exhibit low aqueous solubility, stock solutions should be prepared in 100% DMSO (or the recommended DMSO/buffer for storage), with working dilutions prepared fresh; (iv) Inner-filter effect: Fluorescence quenching due to inner-filter effects, often observed at higher substrate concentrations, must be properly corrected [102]. For high-throughput screening (HTS) assays, short non-FRET based substrates [61], reporter-based designs [91], electrochemical detection [100] and other alternative platforms [98] are cost-effective and highly and easily scalable solutions.

Regarding recombinant expression, Mpro constructs should be selected or designed based on: (i) affinity column and protease availability for protein capture and tag cleavage, respectively; (ii) acquisition of tag-free Mpro containing native termini without additional residues. If a single step purification protocol is required, a 6xHis tag can be placed at the C-terminus [32] or on-column tag cleavage can be performed [58]. Although buffer compositions reported in the literature for Mpro activity assays exhibit considerable variability, standard consensus recommendations include: (i) buffer pH values at near-physiological conditions (6.5–8.0); (ii) including metal-chelating and reducing agents, such as EDTA and DTT/TCEP, respectively. DTT should be avoided when evaluating the inhibition of compounds containing highly electrophilic groups designed to act as covalent warheads; (iii) limiting DMSO concentration to a maximum of 20%, using it only when required for substrate or inhibitor solubility. Finally, for detailed guidelines on kinetic parameter determination and the elucidation of binding modality, we recommend the comprehensive and extensive work by Voget and Gütschow [86].

## Data Availability

No datasets were generated or analysed during the current study.

## Declarations

### Generative AI and AI-assisted technologies in the writing process

In this review, the author(s) responsibly used ChatGPT and Gemini Pro to improve the clarity of the English language.
